# A tale of SARS-CoV-2 genomic surveillance in Mali: Variants introductions and transmission dynamics

**DOI:** 10.1371/journal.pone.0352020

**Published:** 2026-06-25

**Authors:** Noumou Yakhouba Keita, Harris Onywera, Mahamadou Abdou, Zeinaba Dicko, Fankélé Mamadou Diarra, Ousmane Kamena, Amidou Diarra, Klèma Marcel Koné, Alhadji Alassane Dicko, Demba Koita, Tinimba Sountoura, Oumar Samaké, Korika Diakité, Soumahila Traoré, Rabiatou Sanogo, Boubacar Doumbia, Ibrehima Guindo

**Affiliations:** 1 laboratory department, Institut National de Santé Pulique (INSP), Bamako, Mali; 2 Africa Centres for Disease Control and Prevention (Africa CDC), Addis Ababa, Ethiopia; 3 Faculty of Pharmacy, Université des sciences des Techniques et des Technologies de Bamako, Bamako, Mali; Central South University, CHINA

## Abstract

Despite the global scale of SARS-CoV-2 genomic surveillance, data from West Africa are limited. In Mali 33,197 confirmed cases and 743 deaths (case fatality ratio: 2.23%) were recorded between March 2020 and August 2025. This study aimed to provide the first comprehensive genomic reconstruction of SARS-CoV-2 variant introductions, transmission pathways, and evolutionary trends in Mali from 2020 to 2023. A total of 548 Malian sequences were used for lineages assignment and variant tracking. For the reconstruction of transmission dynamic a high quality subset of 305 Malian sequences (passing 90% coverage) was analysed alongside 142 closely related global genomes (from 20 countries) identified using the UShER. Time-resolved phylogenetic reconstruction, ancestral trait inference, and molecular clock analyses were performed with TreeTime v0.11.4 to infer variant introductions, local transmission, and substitution rates. Genomic and epidemiological data revealed five distinct epidemic waves driven by the temporal succession of global variants, from 19A/20A lineages in early 2020 to dominant XBB sublineages in 2023. Multiple independent viral introductions were traced primarily to Europe and North America, followed by sustained local and intercontinental transmission, including reciprocal exchanges with neighbouring African countries. The substitution rate was estimated to be 1.56 x 10−3 substitutions per site per year, consistent with global evolutionary trends. Entropy analysis revealed high genetic variability within the spike gene, particularly in the receptor-binding domain. These findings underscore the persistent global connectivity and local evolutionary pressure shaping the trajectory of the pandemic in Mali. These call for the need for sustained genomic surveillance capacity to inform epidemic preparedness and response.

## Introduction

The emergence of the severe acute respiratory syndrome coronavirus 2 (SARS-CoV-2) in December 2019, triggered an unprecedented global health crisis, with 779 million confirmed cases and 7.1 million deaths reported by February 2026 [1]. SARS-CoV-2 is a positive-sense single-stranded RNA virus with a genome that encodes non-structural proteins (nsp1–16), four structural proteins (membrane (M), envelope (E), nucleocapsid (N) and spike (S)), as well as eight accessory proteins [2,3]. Its proteins involved in nucleic acid replication and repair are of particular importance because they determine the rate of mutations through their degree of fidelity, their ability to correct replication errors and damage to the RNA [4]. The viral genomic RNA is replicated by RNA-dependent RNA polymerase (RdRp) [5]. Unlike most viral RNA polymerases which do not possess proofreading and correction activity, viruses of the order Nidovirales to which the Coronavirus genus belongs, possess complex molecular machine composed, among others, of RdRp (also called nsp12) and an exonuclease (nsp14) which corrects nucleotide incorporation errors of RdRp [6–8].

Despite the proofreading activity of nsp14 and its cofactor nsp10, over the years, SARS-CoV-2 has accumulated mutations [9,10]. Mutations within the S protein are of particular interest because of their implications on transmissibility and immune escape [11,12], and they underpin the emergence of SARS-CoV-2 variants. The impact of the mutations on disease severity, transmission, diagnostics, immune and therapeutic escape, determine the classification of variants into variants of interest (VOI), under monitoring (VUM) or of concerns (VOC) [13–15]. Variants emerged with the global spread of SARS-CoV-2. In September 2020, the first variant of concern, Alpha (B.1.1.7) was reported in United Kingdom harbouring seventeen mutations among which eight in the S protein. In addition to the N501Y mutation carried by the Alpha variant, the Beta (B.1.351) variant was reported in South Africa with two additional mutations (K417N and E484K) in the receptor-binding domain (RBD). These mutations collectively enhanced receptor binding affinity, transmission, and immune escape [16,17]. The emergence of the Delta VOC in India December 2020, was associated with increased transmission and mortality [16]. Since, its emergence in November 2021, the Omicron (B.1.1.529) and sublineages represented 98% of the sequenced deposited in public database by February 2023, warranting the update of the World Health Organization (WHO) SARS-CoV-2 variants tracking system [18].

Global genomic surveillance strategy and initiatives have been promoted to track the evolution of the virus and share genomic and metadata under the FAIR (Findable Accessible Interoperable Reusable) principles [19,20]. Early in the Coronavirus disease 2019 (COVID-19) pandemic, global sequencing efforts were uneven, with limited representation from Africa and other low- and middle-income regions [21]. This constrained a comprehensive understanding of SARS-CoV-2 transmission and evolution. However, substantial investment, capacity-building efforts, and collaborations across the continent have since transformed this landscape, thereby strengthening pathogen genomic capacity and enabling more timely variant tracking, regional phylogeographic analysis, and rapid public health responses. This progress is a testament to the expansion of in-country sequencing capacity and the achievement of shorter turnaround times for data generation and sharing [22].

In Mali, the first two COVID-19 cases were reported on 25 March 2020 [23]. By August 2025, 33,197 cases have been registered with 743 deaths [1]. Similar to many other countries, the epidemic in Mali has been characterized by five epidemiological waves and the emergences of variants [24]. Preliminary genomic sequencing analyses of 21 specimens reported the circulation of 19A and 20B in April 2020 [25] and later (April to October 2021) detection of five additional variants among which three VOCs (Alpha, Beta and Delta) [26]. Through genomic capacity building initiatives, such as African Pathogen Genomics Initiative and the Afroscreen Project, genomic data have been generated and partly deposited to the global initiative on sharing avian influenza data (GISAID) [27,28]. Despite report on variants circulation, the timing, origin, and evolutionary patterns of SARS-CoV-2 variant introductions and subsequent local spread remain poorly described. Understanding these patterns helps explain how outbreaks spread, informs border health measures, and supports regional readiness for new respiratory threats. We aimed to infer the introduction and spatiotemporal distribution of SARS-CoV-2 variants in Mali by analyzing publicly available genomes complemented by unpublished data generated at the Institut National de Santé Publique (INSP), thereby providing the first comprehensive genomic account of the country’s epidemic from 2020 to 2023.

## Methodology

### Mali surveillance system

Situated in West Africa, Mali occupies more than 1.2 million square kilometer with around 24 million habitants [29]. The strategic COVID-19 laboratory response plan designated four laboratories meeting the biosafety and technical requirement for molecular diagnosis under the coordination of the INSP. Specimens collected from districts and hospitals across the country were initially sent to INSP and distributed to the designated laboratories for testing. Diagnosis has then been decentralized and extended to point of care available at district level with GenXpert. The results were centralized at INSP and reported to the ministry of health for daily communication. The temporal framework of this study encompasses two distinct periods: a longitudinal epidemiological surveillance from 2020 to August 2025 and a genomic surveillance timeline (2020–2023) when 99.90% of cases were detected.

### Molecular detection of SARS-CoV-2 at INSP

RNA was extracted from naso-oropharyngeal swab with QIAamp Viral RNA Mini Kit (Qiagen Inc, Valencia, CA, USA) and MagMAX Viral/Pathogen Nucleic Acid Isolation Kit on Kingfisher flex (Thermofisher, USA) following manufacturer instructions. RTqPCR were performed with TaqPath™ COVID-19 CE-IVD RT-PCR Kit on QuantStudio™ 5 (Applied BiosystemsTM, California, USA) following manufacturer instructions.

### Specimen sampling for whole-genome sequencing

Specimen with cycle threshold (ct) value below 30 were eligible for whole-genome sequencing (WGS) based on convenience sampling strategy. During the study period, INSP acted as a central diagnosis hub processing approximatively 85% of all SARS-CoV-2 samples in Mali. More importantly, testing for international travellers were exclusively performed at INSP, representing between 70–85% of daily testing volume. Prior to WGS capacity development, 104 specimens covering 2021 and the first quarter of 2022 were sequenced at WHO collaborative center “Institute Pasteur de Dakar (IPD)” in Senegal, while 100 specimens were sequenced at CHU Henri Mondor France through a training program organized by the Afroscreen Project. A total 212 specimens were sequenced at INSP covering 2020–2023. All samples submitted to whole-genome sequencing were archived samples. The data were anonymized and accessed by the authors on December 31^st^ 2025. The ethics committee approval was obtained under N°2025/259/CE/USTTB.

### Library preparation and sequencing

#### Oxford Nanopore Technology.

The library was prepared with the Midnight RT Expansion Kit (Oxford Nanopore Technology, Oxford, UK) following manufacturer recommendation. Briefly, following, retrotranscription, two primer pools were used to generate 1200-base pair (bp) tiled amplicons. The amplicons were barcoded with 1 µL of rapid barcode, pooled and purified with Ampure XP beads (Beckman Coulter, California, USA). The rapid adapter was added to 800 ng before loading, sequencing and basecalling on Minion MK1C (Oxford Nanopore Technology, Oxford, UK) [30].

#### Illumina.

The Illumina COVIDSEQ Assay (Illumina, California, USA) was used for library preparation under manufacturer instructions. Following cDNA synthesis, the Artic Network primer v4 was used to generate 400-bp amplicons. The amplicons were indexed, purified and quantified on Qubit 4 Fluorometer (Thermofisher, Maryland, USA). The normalized library was loaded on the NextSeq 550 for library sequenced at CHU Henri Mondor and IPD and on NextSeq 2000 (Illumina, California, USA) for those sequenced at INSP [31].

### Consensus sequence generation

The consensus files were generated on EDGE COVID-19 for library sequenced at IPD, whereas EPI2ME Artic workflow and the bacterial and viral resources center (BVBRC) were used for reads generated at INSP [32–34]. On EDGE COVID-19 the default parameters for Illumina reads were applied starting with FaQCs v2.09 for quality control and trimming below 20 quality score and 50 read length followed by primer trimming [35]. The trimmed reads were mapped to the SARS-CoV-2 (NC_045512.2) reference sequence with bwa v0.7.12 while SAMtools v1.10 and BCFtools v1.10.2 were used for variant calling and consensus generation [36–38]. For reads analyzed on EPI2ME, the Artic workflow was executed which utilize FastQC for quality control minimap2 v2.18 for mapping reads to reference and medaka v1.7.2 for consensus calling [39–41]. Finally, reads analyzed on BVBRC followed a similar workflow with exception of adapter trimming with cutadapt and read mapping with bowtie2 [42,43]. The lineages were assigned with nextclade v3.8.2 [44].

### Bioinformatic analysis for variants introduction and transmission dynamic

**Fig 1:**
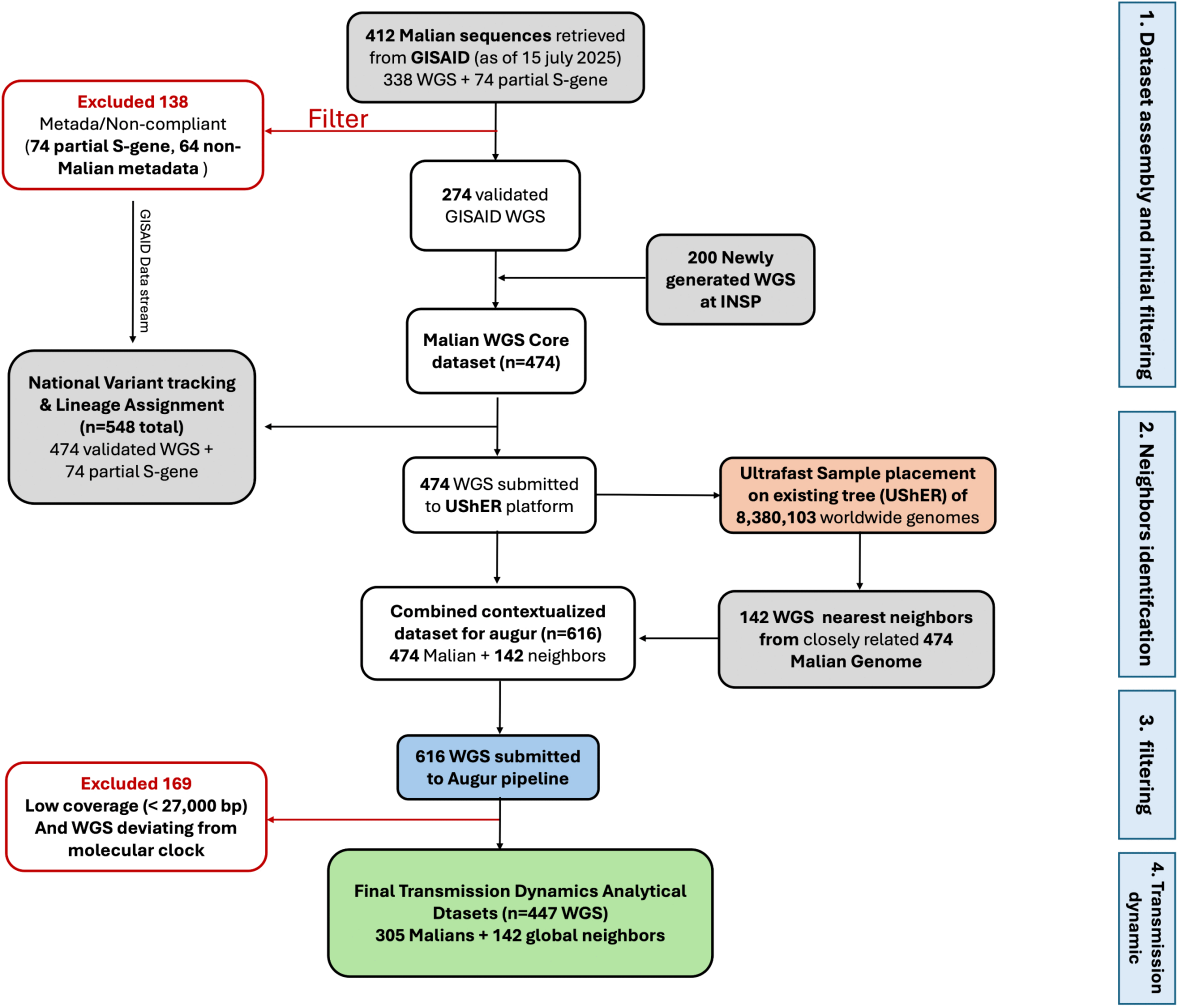
Data preprocessing and analytical pipeline. The flowchart illustrates the harmonization of public data retrieved from GISAID and local generated WGS from INSP. The sequences were submitted to two tier filtering: (1) metadata filtering and QC for national variant tracking (n = 548) and (2) stringent coverage filtering (>27,000 bp) for high resolution transmission dynamic data. Through UShER placement, global contextual sequences (n = 142) were identified to provide phylogenetical rooting for the final dataset analytical dataset (n = 447).

**Fig 2:**
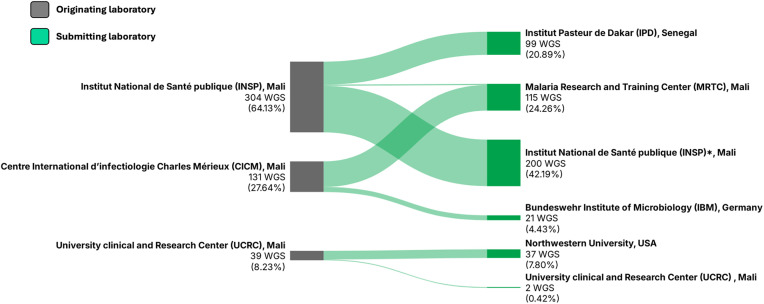
Originating and submitting laboratories contribution to whole-genome sequences sharing on GISAID. *Submission to GISAID on progress.

The analytical workflow was executed following the five steps described below;

- Dataset assembly and placement; A total of 474 whole-genome sequences (WGS) comprising 274 retrieved on GISAID on 15 July 2025 and 200 newly generated WGS at INSP were submitted to Ultrafast Sample placement on Existing tRees (UShER) [45]. UShER nearest neighbour algorithm identified 142 closely related WGS from a global tree of 8,380,103 genomes from Genbank, CoG-UK and China National Center for bioinformatics (25-07-27). This approach ensure that the contextual sequence is the most likely ancestor or descendant of the variants detected in Mali providing context for differentiation of viral introductions and local transmission.
- Quality filtering: to ensure high resolution molecular clock and transmission dynamic analysis, augur v31.3.0 was used to filter the combined dataset (n = 612) by removing any sequences below 27,000-bp (< 90% coverage) [46]. This threshold was used to minimize phylogenetic noise and ensure the accuracy of temporal signal and ancestral traits, as low coverage sequences can lead to branch length inaccuracies in molecular clock models. This resulted in a final analytical dataset of 447 WGS used for transmission dynamics.
- Alignment and masking: Alignment was performed with MAFFT v7.526 followed by masking 35 nucleotides at the 3 prime Untranslated region (UTR) region [47] to remove low confidence phylogenetic signal.
- Phylogenetic inference:. Maximum likelihood (ML) phylogenetic tree was built with IQ-TREE v3.0.1 using the General Time Reversible (GTR) substitution model [48].
- Temporal and trait inference: TreeTime v0.11.4 was used to transform ML tree into time-resolved tree with stochastic resolve mode, infer ancestral traits and and reconstruct transmission trait. The built available on Nextstrain was visualized with auspice v2.66 [49]. The transmission vectors were extracted from auspice and blent on natural earth vector data projected in web mercator (EPSD:3857). The analytical workflow and WGS origins are respectively described in Figs 1 and 2.

### Ethics statement

The study was conducted in accordance with the Declaration of Helsinki. The study was approval was the ethics committee of the faculty of medicine and pharmacy of the university of sciences, techniques and technologies of Bamako (USTTB) under the number N°2025/259/CE/USTTB.

## Results

### Epidemiological situation

**Fig 3:**
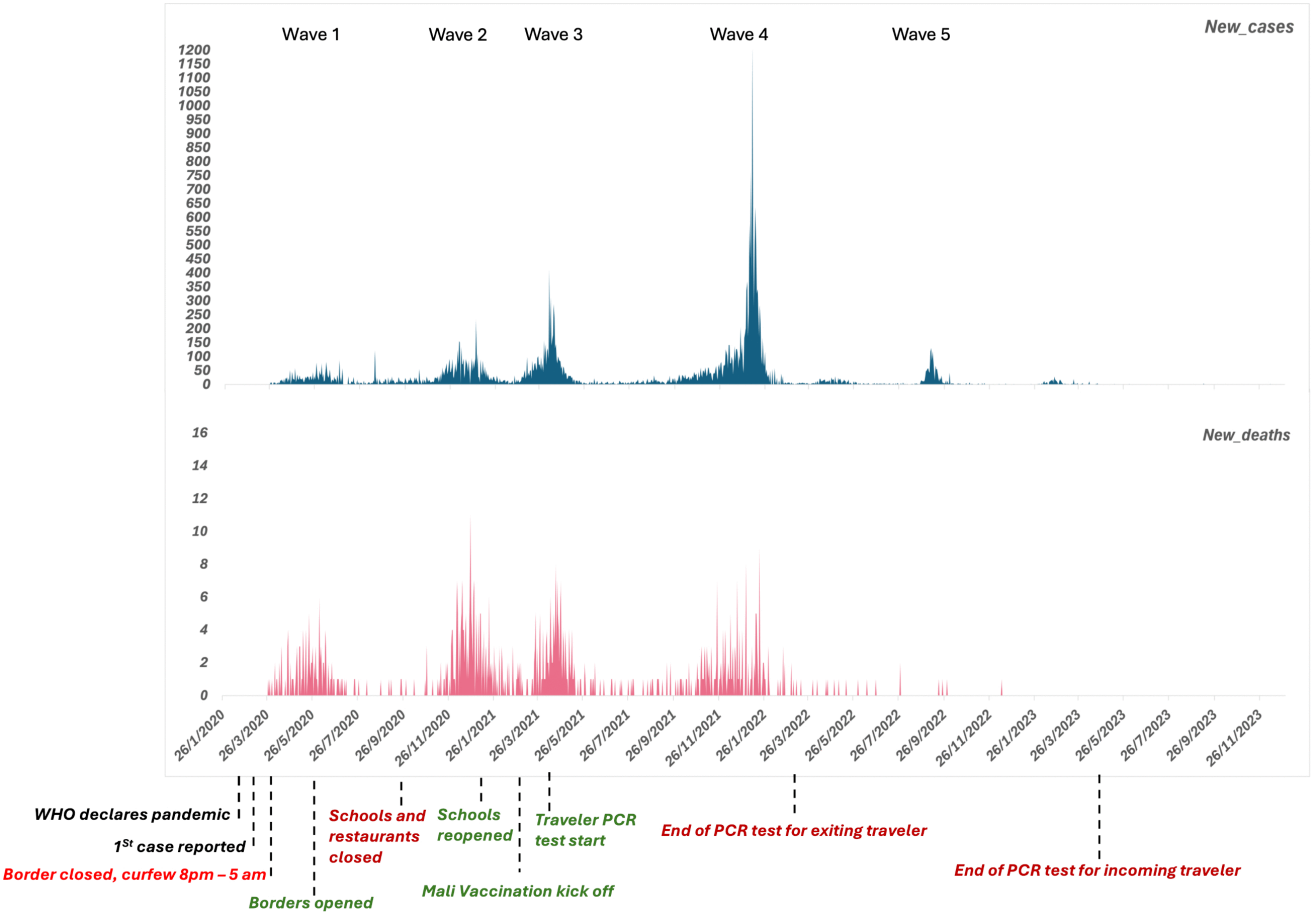
Epidemiological curve and public health interventions in Mali. The top panel (blue) represent daily laboratory confirmed cases, while the bottom panel (pink) represents daily recorded death. Data shown covered the period between the first case and 31 December 2023, as only19 and 14 cases have been reported in 2024 and by August 2025, respectively.

Given the evolving patterns of COVID-19 transmission and mortality in Mali, this study aimed to characterize the epidemic trends and investigate the genomic diversity of circulating SARS-CoV-2 variants. Between March 2020 and August 2025, Mali recorded 33,197 confirmed COVID-19 cases and 743 deaths, yielding a case fatality ratio (CFR) of 2.23%. Five distinct epidemic waves were observed with varying CFR (Fig 3). while longitudinal monitoring continued through august 2025, the majority (99.90%) of cases were reported before the end of 2023. Following the first cases, borders were closed and reopened after the first wave. The relative few numbers recorded in this period motivated the lockdown of restaurants, schools and public spaces at the beginning of the second wave with limited impact on disease transmission. The second (CFR: 4.01%) and third (CFR: 3.02%) waves exhibited the highest fatality despite lower cases counts, while the fourth wave (September 2021 to February 2022) accounted for nearly 50% of all reported cases (15,505) but a lower CFR (1.18%). The decline in fatality during later waves coincided with expanded diagnostic capacity, vaccination rollout, and circulation of less virulent Omicron sublineages. These temporal variations indicated underlying changes in viral characteristics, population immunity, and public health interventions that warranted genomic interrogation.

### Relative temporal variants distribution

**Fig 4:**
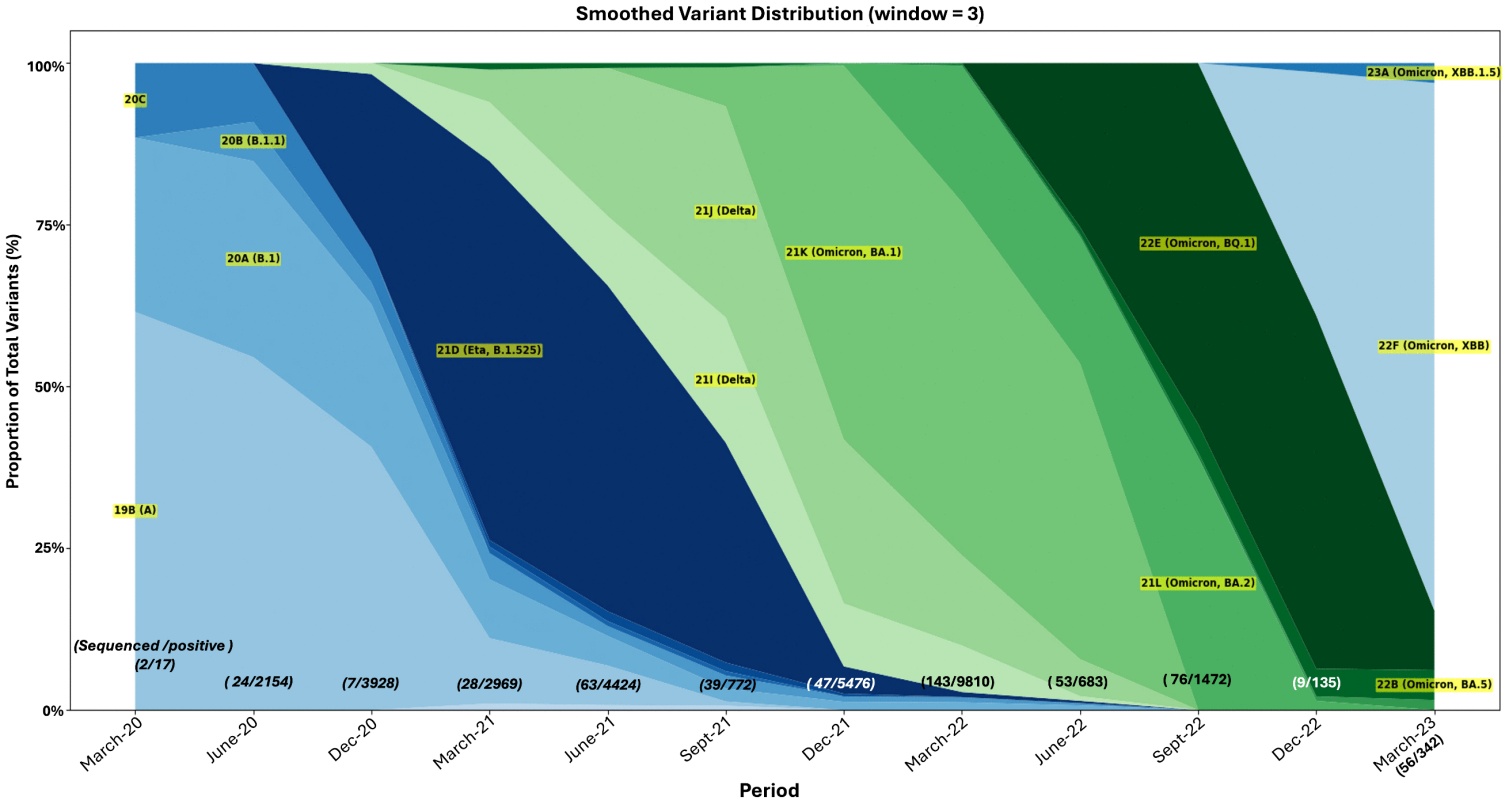
Smoothed relative temporal distribution of SARS-CoV-2 variants in Mali. The graph was generated with a custom python script using matplolib and pandas libraries. The data was binned by month and a 3-month rolling window was applied to smooth transition between variants to provide a clear visualization of temporal succession of clades. The sequencing rate (number of samples sequenced/positive samples) is given per period.

Building on the observed temporal variations in case burden and fatality, we analyzed SARS-CoV-2 genomic data to assess the diversity, lineage distribution, and variant transitions in Mali from 2020 to 2023. During this time, a total of 548 samples were sequenced, representing 1.65% of the confirmed cases. The relative temporal distribution of variants depicts the circulation of 19A and 20A in the early months of the epidemic in Mali in 2020 followed by the emergence of Alpha and Beta variants (Fig 4). The 21D (Eta B.1.525) variant dominated the first semester of 2021 before the circulation of two Delta variants and their sublineages and the rise of ancestor of the Omicron variant, 21M (B.1.1.529). In 2022, all variants detected onward were sublineages of 21M, with 21k (Omicron, BA.1) leading before being overtaken by 21L (BA.2). Two descendants of BA.2 were circulating in 2023, 22B (BA.5) and 22F (XBB) with the later one and its sublineages dominating.

### SARS-CoV-2 variants introductions and transmission dynamic

**Fig 5:**
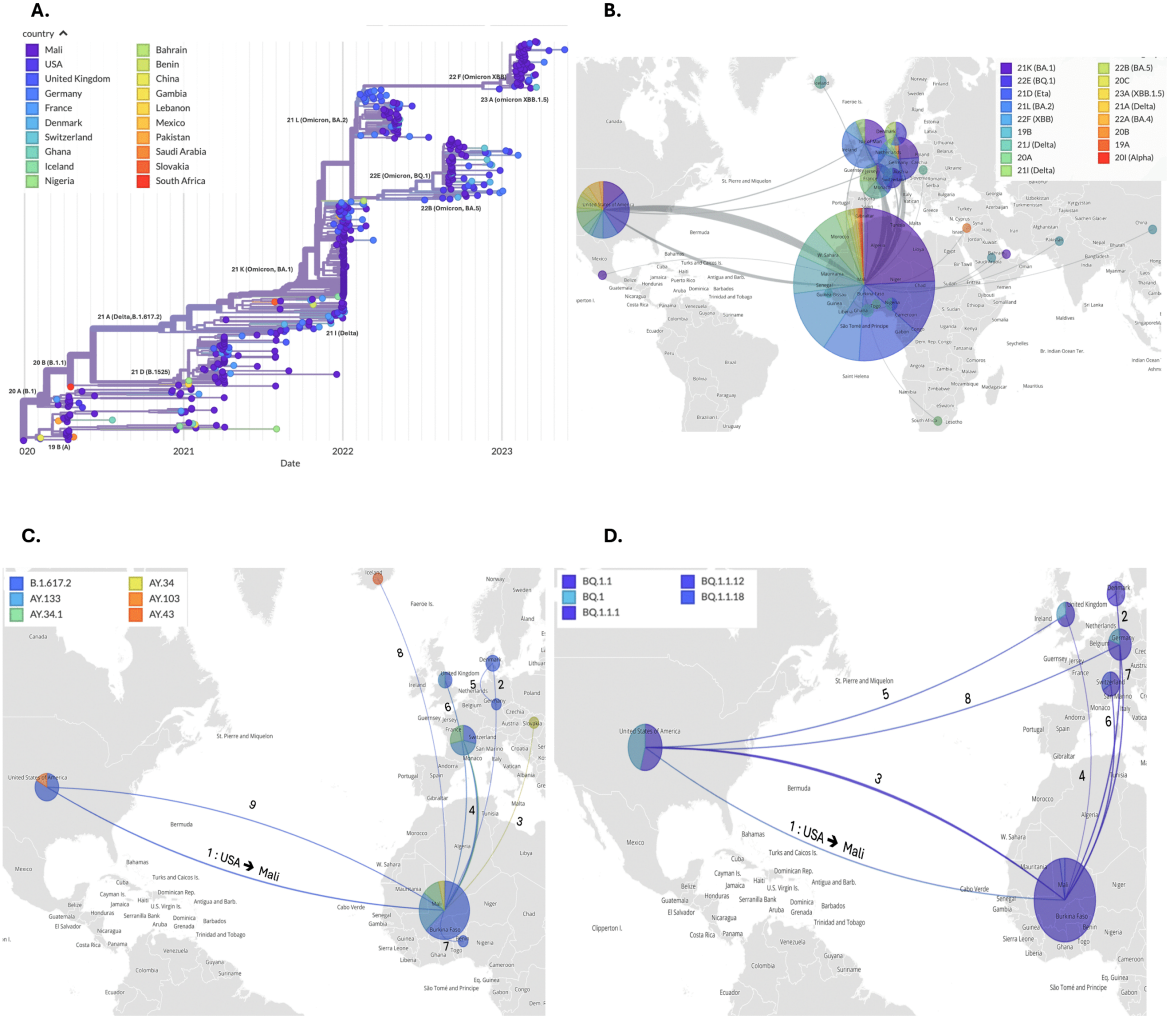
SARS-CoV-2 variants introduction, evolution and transmission dynamics. **A)** Phylogenetic tree depicting genetic divergence between of SARS-CoV-2 variants detected in Mali and other countries. **B)** SARS-CoV-2 transmission line, thick line represents more observed cases movement between two locations. Transmission direction is inferred by the orientation of the arc, from source to origin location, the arc is positively oriented that is the curve is on the left side. C) delta variant transmission dynamic with number describing the events order. **D)** BQ.1 variant transmission dynamic depicting reciprocal transmission between USA and Germany prior to introduction in Mali from the USA followed by transmission with European countries. Map base layers were sourced from Natural earth (public domain) and generated on QGIS v4.0.0.

To investigate spatiotemporal spread, we constructed a time-resolved tree using genomic data from Mali and closely related genomes (Fig 5A). SARS-CoV-2 was first introduced in March 2020 by travelers from France. The genomic data inferred introductions of 19B (A) from China and 20A (B) from United States of America (USA). These events triggered local transmission within Mali and reciprocal spread with Ghana, Senegal and Nigeria, leading to the emergence of further sublineages. Notably, intercontinental transmission by 20A (B) from Mali to Asia (Pakistan) and middle east (Saudi Arabia) was also observed.

By the second quarter of 2020, a second wave of independent introductions occurred from France, USA and South Africa. This period was characterized by the circulation of B.1 with USA strain being the most recent common ancestor of all variants detected onward. Local B.1 transmissions later gave rise to the 20I (Alpha, B.1.1.7) and the 21D (Eta, B.1.525) variants. These variants exhibited high rate of reciprocal transmission between Mali and Europe, particularly with France, England and Germany. (Fig 5B).

The ancestor of the Delta variant was introduced in Mali from USA in the last quarter of 2020 leading to its emergence around April 2021. Different lineages of Delta circulated before the circulation of 21K (Omicron, BA.1) in the last semester of 2021 (Fig 5C). Two clades dominated the first semester of 2022, 21L (Omicron, BA.2) introduced simultaneously from Germany and UK and 22B (Omicron, BA.5) from USA. The end of 2022 was marked by the circulation of 22E (Omicron, BQ.1), originating from USA followed by circulation between Mali and Europe (Fig 5D). The last detected variant, 23A (Omicron, XBB.1.5), was preceded by 22F (Omicron, XBB), which was introduced in December 2022 from USA, with local transmission leading to clusters in the first quarter of 2023.

### SARS-CoV-2 molecular clock and genetic diversity

**Fig 6:**
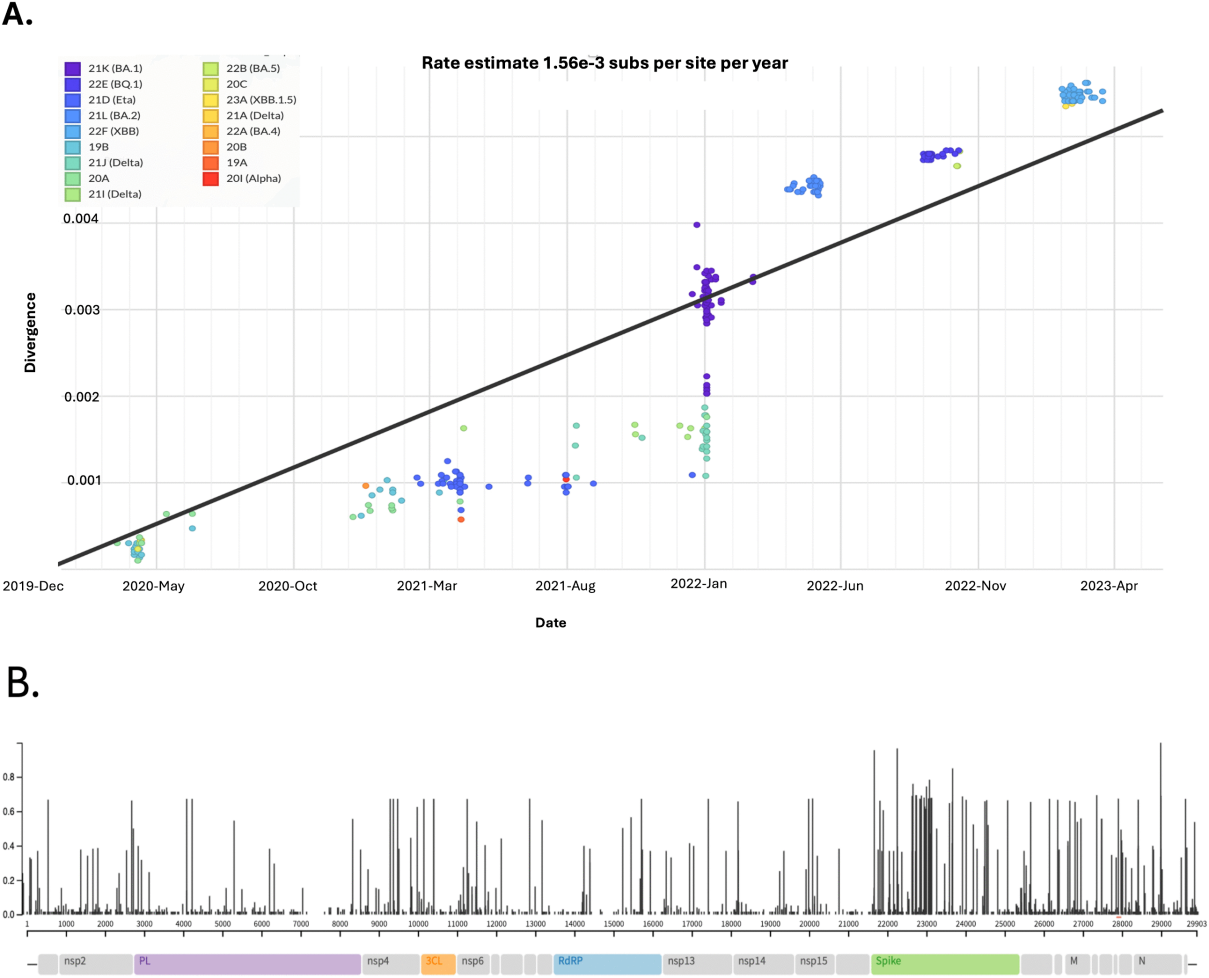
Genetic diversity of Malian strains. **A)** Molecular clock describing strain divergence on the y-axis per unit time (x-axis) colored by clade. **B)** Entropy (genetic diversity per genomic position). The spike protein in green displayed high diversity with entropy as high as 0.92 for certain position.

To gain insights into the evolutionary rate and mutational landscape of SARS-CoV-2 in Mali, molecular clock and entropy analyses were performed on high-quality WGS. The substitution rate of the build was estimated as 1.57 x 10−3 substitutions per site per year, which slightly decreased to 1.56 x10-3 when filtered for Malian strains (Fig 6A). Considering a genome size of 29,903 nucleotides, this corresponds to an accumulation of approximately 47 new mutations per genome every year. Entropy analysis revealed significant genetic variation across the genome with multiple regions of high genetic diversity. As shown in Fig 6B, high entropy values were particularly observed in the S protein, especially within RBD. Notably, positions 28,881 in the N gene exhibited high entropy among all genomic sites.

## Discussion

This study presents the first comprehensive genomic reconstruction of SARS-CoV-2 variant introductions, transmission dynamics, and evolutionary trends in Mali from 2020 to 2023, thereby closing a major gap in West Africa’s COVID-19 evidence base. Through the combined analysis of local and global genomic data, this study uncovered how cross-border viral flow, variant succession, and local epidemic pressures influenced the course of COVID-19 in Mali. The sustained circulation of SARS-CoV-2 in Mali between 2020 and 2023, characterized by five distinct epidemiological waves, provides insight into the long-term dynamics of the pandemic in the country. The overall CFR of 2.23% is slightly higher than the continental average of 1.8%, comparable to rate reported by neighboring countries such as Senegal (2.21%) and lower in other places like Ivory Coast (0.9%) [1]. The cumulative CFR masks temporal heterogeneity. The pronounced difference in CFR between high-volume fourth wave (1.8%) and lower volume but more lethal second (4.1%) and third (3.02%) waves may be indicative of underlying shifts in viral characteristics, healthcare capacity and/or population immunity over time. In fact, the high mortality observed in the early waves could reflect a period of low population immunity prior to vaccine availability, limited testing due infodemic, and isolation or quarantine imposed on confirmed cases. The lethality difference may also reflect a shift in intrinsic viral characteristics, from highly virulent, less transmissible variants to less virulent, more transmissible ones, exemplified by the sequential circulation of Alpha, Beta, Eta and Delta in the first three waves while, the fourth was dominated by Omicron and residual Delta circulation [50]. In fact, the mortality ratio associated with the Alpha (B.1.1.7) variant was 2.26 times higher than that of preceding strains [51]. Moreover, studies on the risk posed by newly emerging SARS-CoV-2 variants have shown that Omicron subvariants evolved to trade reduced immunopathogenesis for increased angiotensin-converting enzyme 2 (ACE2) binding [52,53].The decentralization of diagnostic capacity, through wider use of rapid antigen and point-of-care molecular tests, may also have contributed to the number of reported cases.

Despite, a relatively low sequencing coverage (1.65%), genomic surveillance in Mali successfully captured the major global variant successions, establishing the country as an active participant in the global genomic surveillance landscape. The sequential detection of lineages 19B and 20A, followed by the emergence and subsequent replacement of Alpha, Beta, Eta, multiple Delta lineages, and the dominance of the Omicron lineages (BA.1, BA.2, BA.5, and XBB), confirms viral evolution fueled by repeated introductions and sustained local and international transmission. The limited number of early sequences infers introductions of 19B and 20A from China and USA, respectively followed by local and intercontinental transmission despite border closures.

The dominance of 21D (Eta, B.1.525) in the first half of 2021, before the global surge of the Delta variant is noteworthy. This variant, which seems to emerge in late 2020, was detected in all continents by March 2021 [54]. In Mali, it represented 32 of 36 of genomes deposited since its emergence through May 2021 [55]. The Eta variant, considered a VOI because of its latent decrease in neutralization by antibody and vaccine-induced sera, harbours several mutations (E484K, D614G, A67V, Δ69–70, etc) found in Alpha, Beta, and Gamma variants along with E483K mutation and a novel F888L mutation in the S2 domain of the S protein [50]. These mutations likely favoured its fitness, enabling Eta variant to replace Alpha and Beta in the first quarter of 2021. The circulation of the Eta variant was sustained in Mali through reciprocal transmission with European and other African countries.

The Delta variant first reported globally in December 2020, was detected in Mali around April 2021 and subsequently displaced the Eta variant in Mali. As reported globally, Delta lineages dominated until the last quarter of 2021, spanning the third and fourth waves in Mali [56]. The Delta variant globally recognized for its increased transmissibility and severity, was temporarily associated with the more lethal third waves (CFR: 3.01%) [57]. It harbours several key mutations, L452R and P681R, also present in the Kappa (B.1.617.1) variant [50,58], which could partly explain its increased lethality compared to preceding variant. The L452R mutation is thought to increase ACE2 binding affinity and aid escape from attack by CD8 + T-cells, while P681R is thought to improve fusion and integration into the host cell [59].

The Delta variant was subsequently displaced by the 21K (Omicron, BA.1) at the end of 2021, like due of its higher infectivity. This variant is predicted to be approximately 10 times more contagious than the Wuhan-Hu-1 strain and 2.8 times more transmissible than Delta variant, primarily due to N440K, T478K, and N501Y mutations in the RBD [60]. Following the detection of 21K (Omicron, BA.1), it was overtaken by the BA.2 variant. In vitro studies have inferred that BA.2 is 1.5 times more contagious than BA.1 and exhibits significantly better cell fusion [61]. This pattern of rapid variant displacement continued with the circulation of BA.5, introduced from the USA. Reciprocal transmission of the 22E (Omicron, BQ.1) ancestor between USA and Germany was observed in late 2022 before its introduction into Mali from the USA, demonstrating complex multi-step intercontinental transmission pathways. In December 2022, the 22F (Omicron, XBB) variant, thought to have emerged from recombination between two of BA.2 lineages, was introduced in Mali from USA. Its rapid local transmission and subsequent dominance throughout 2023, along with its sublineages, reflect the global trend of XBB’s strong fitness advantage, particularly its ability to evade immunity from prior infection and vaccination. Studies have implied the role Y144del and F486S in immune escape, while V83A and N460K are thought to enhance infectivity [53]. The most recent variant detected 23A (Omicron, XBB.1.5), emerged from XBB.1 and carries these additional mutations, S:G252V, S:F486P, and ORF8:G8stop mutation [62]. These successive Omicron introductions demonstrate the high genetic fluidity and sustained viral exchange between Mali and major travel hubs, particularly North America and Europe.

The estimated substitution rate of 1.56 x 10−3 per site per year, confirms the consistent evolutionary rate of SARS-CoV-2 in the Malian context, corresponding to approximatively 47 new mutations per genome annually. The observed substitution rate, which is approximately 1.7 times higher than the global rate (27.68), could be explained of dominance of rapidly evolving variants in the build [63]. In fact, sequencing capacity development coincided with the circulation of Delta and Omicron variant, which represent 71.1% of available whole-genome sequences. This sustained accumulation is reflected in the noticeable genetic diversity, particularly high in the RBD of the spike gene. The high entropy in RBD corresponds to the expected region of intense evolutionary pressure for immune escape and enhanced ACE2 receptor binding. High entropy was observed at nucleotide position 28,881 in the N gene. The Wuhan strain and early variants carried a guanine (G) while the latest variant carried an Adenine (A). At amino acid level, this result in the R203K mutation, associated with increased infectivity, fitness, and virulence [64].

The primary limitation of this study is the low sequencing genomic coverage (1.65%), although the available sequencing data successfully captured major evolutionary dynamics. This implies that some local transmission clusters and minor variants’ introductions may have been missed. Future efforts should focus on scaling up sequencing capacity and systematic sampling to improve detection sensitivity. Despite the limitation, this longitudinal genomic analysis from Mali describes a three-year pattern of sequential variant circulation, fluctuating CFR, and sustained viral exchange, thus case fatality heterogeneity, therefore emphasizing the need for ongoing genomic surveillance to strengthen epidemic preparedness in African countries and beyond.

## Conclusion

This study provides the first longitudinal genomic reconstruction of SARS-CoV-2 variant introductions and transmission dynamics in Mali from 2020 to 2023. We found that the epidemic was shaped by recurrent international introductions, sustained community transmission, and variant replacement patterns that mirrored global evolutionary trends. Multiple importation events from Europe and North America, as well as bidirectional exchanges across West Africa, demonstrated that regional and global connectivity continued to influence the epidemic even during restricted mobility. The diversity within the spike RBD indicated ongoing viral adaptation and immune selection. These findings call for strong, locally led genomic surveillance to detect emerging variants early, guide effective interventions, and prevent cross-border spread. We echo Africa CDC’s New Public Health Order, which emphasizes that robust public health security in Africa, including in Mali, requires sustained investment in genomic surveillance, infrastructure, and workforce development [65].

## Supporting information

S1 File Malian and contextual fasta sequences used in this study for lineage tracking and transmission transmission dynamic. (FASTA)

S2 Table Metadata of the Fasta sequences. (XLSX)

S3 File Augur build for transmission dynamic and genetic diversity visualization. (JSON)
